# Training for and Dissemination of the Nutrition Environment Measures Surveys (NEMS)

**Published:** 2010-10-15

**Authors:** Sally Honeycutt, Erica Davis, Margaret Clawson, Karen Glanz

**Affiliations:** Department of Behavioral Sciences and Health Education, Rollins School of Public Health, Emory University; the Schools of Medicine and Nursing, University of Pennsylvania, Philadelphia, Pennsylvania; Rollins School of Public Health, Emory University, Atlanta, Georgia, and the Schools of Medicine and Nursing, University of Pennsylvania, Philadelphia, Pennsylvania; the Schools of Medicine and Nursing, University of Pennsylvania, Philadelphia, Pennsylvania

## Abstract

**Introduction:**

Researchers believe that *nutrition environments* contribute to obesity and may explain some health disparities. The Nutrition Environment Measures Surveys (NEMS) are valid and reliable observational measures of the nutrition environment. This article describes the dissemination of the measures, including the development, implementation, and evaluation of training workshops, and a follow-up survey of training participants.

**Methods:**

To disseminate the NEMS measures, we developed a 2-day intensive, participatory workshop. We used an immediate postcourse evaluation and a structured telephone follow-up interview to evaluate the workshops and the dissemination strategy. Topics included use of the NEMS measures, reactions to the workshops, and participants' training others on the measures.

**Results:**

During the study period, 173 people participated in 14 workshops. Participants indicated a high level of satisfaction with the training workshops. Almost two-thirds of respondents reported using the measures to train an additional 292 people and to rate more than 3,000 food outlets. The measures have been used in diverse locations across the United States for various purposes. Respondents have reported NEMS results in peer-reviewed journals, master's theses, newspaper articles, and presentations.

**Conclusion:**

The NEMS measures are the only nutrition environment measures that have been packaged for distribution and widely disseminated. The measures fill a need in the worlds of research and community action, and dissemination was successful in accelerating diffusion and promoting adoption of the measures. The use of an ongoing, continual process to improve workshops and measures contributes to the usefulness of the surveys and accelerates their adoption and continued use.

## Introduction

The prevalence of obesity, a risk factor for many chronic diseases, is increasing in the United States (1-3). *Nutrition environments* — the social, policy, and built environments that influence access to food — may contribute to obesity and may explain some disparities in health behaviors and outcomes (4-8). A conceptual model of nutrition environments depicts multiple levels, including *community* nutrition environments (ie, the number, type, location, and accessibility of food outlets) and *consumer* nutrition environments (ie, the availability and price of, and information about foods in those outlets) (5). Researchers, policy makers, and obesity prevention program managers have shown an increasing interest in understanding and assessing the nutrition environment and other nutrition-related issues (6).

McKinnon and colleagues (6) conducted a literature review of nutrition environment measures reported during 1990 to 2007. They identified 137 articles with measures of food environments. Most used interviews or questionnaires rather than observation, and few (only 13%) provided any information on reliability or validity of the measures (6). The Nutrition Environment Measures Surveys (NEMS) were developed to address the emerging need to better assess and understand nutrition environments (9,10). These surveys provided the first reliable and valid observational measures of community and consumer nutrition environments (6,9). The NEMS measures consist of 2 surveys, 1 for use in stores (NEMS-S) and 1 in restaurants (NEMS-R). Trained raters use the surveys to observe and rate food outlets. NEMS-S rates price and availability of 10 indicator food categories and assesses quality of fresh fruits and vegetables (9). NEMS-R assesses the availability of healthy regular and kids' menu options, facilitators and barriers to healthy eating, and prices (10). Measures, protocols, and a description of the development process have been reported previously (9,10). Both surveys have high interrater and test-retest reliability, and the measures were found to have both face and construct (discriminant) validity; they confirmed hypothesized differences in the availability of healthy options in grocery stores versus convenience stores and low- versus high-income neighborhoods (9,10). When interviewers are adequately trained and quality control is maintained, the surveys provide valid and reliable observational measures of the consumer and community nutrition environment.

Standardized use of such measures may strengthen research on the effects of nutrition environments on individual behaviors, inform interventions, and shape public policy (6). However, most research-tested innovations are never widely used (11-15). The diffusion of innovations model provides a framework for bridging the gap between research and practice (11,16). This model suggests that multiple types of knowledge about an innovation (awareness, procedural, and principles) influence adoption. Characteristics of the innovation that promote diffusion include *relative advantage* (offering an improvement over existing options), *compatibility* (fit with audience), *complexity* (being easy to use), *trialability* (being able to be tested before being adopted), and *observability* (having visible, measurable results) (11).

To accelerate diffusion of the NEMS measures, we developed and promoted a training workshop. This article describes the dissemination of the NEMS measures, including the development, implementation, and reach of the workshops, and presents findings from a follow-up evaluation of participants' adoption of the measures.

## Methods

### NEMS workshops

To disseminate the NEMS measures and ensure their appropriate use, we developed a 2-day intensive training workshop with the goal of teaching participants to become proficient at completing the measures. We also offered an optional half-day train-the-trainer workshop. The training format and materials were designed to accelerate diffusion and promote adoption of the measures. The basic workshop consists of 8 sections that address both *principles knowledge* (eg, conceptual framework, overview of the NEMS study) and *procedural knowledge* (eg, how to complete the NEMS-R and NEMS-S measures). Participants received a user-friendly manual and a CD-ROM with all materials saved in modifiable formats. Users are encouraged to modify the measures as needed to fit their study design or priority population. We offered guidance on how to make modifications while retaining the integrity of the measures and how to test the reliability and validity of adapted measures on a small scale.

Consistent with principles of Adult Learning Theory, the NEMS workshop is skill-based, highly participatory, and provides learners with immediate opportunities to apply new skills and information (17-20). Fieldwork allows participants to rate actual stores and restaurants, applying and refining knowledge learned in the classroom. The training team is responsive to feedback from participants and has incorporated participant suggestions to strengthen the workshop. Newly developed materials, including customized measures provided by NEMS users, are available to participants on a password-protected Web site.

We used various communication channels to raise awareness of the NEMS measures and promote the workshops to researchers and practitioners: an NEMS Web site (www.med.upenn.edu/NEMS), announcements on relevant listservs, distribution of brochures at public health conferences, presentations at professional meetings, and word of mouth. The NEMS team provides information about training opportunities in response to requests about the measures and their use. We also provide in-person or telephone support to people who completed the NEMS train-the-trainer workshop and are hosting their own training event.

### Evaluation of NEMS training workshops

To better understand the impact of the NEMS training workshops and their effectiveness as a dissemination strategy, we evaluated 14 workshops held between March 2006 and January 2008. We included all workshops conducted at least 6 months before data collection to allow participants adequate time to begin using the measures. A total of 173 participants attended these workshops. The evaluation had 2 components: postworkshop course evaluations and a follow-up survey. We obtained data on workshops and participants from registration records and other program documents. This evaluation was determined to be exempt from review by the Emory University institutional review board.

At the conclusion of the training workshops, participants (N = 164) were asked to complete a postcourse evaluation. Participants from one workshop (n = 9) did not receive postcourse evaluations. The evaluations included 12 questions assessing various aspects of the workshop (eg, organization, materials, quality of instruction) on a scale of 1 (low) to 5 (high). The course evaluation also included 6 open-ended questions (eg, workshop strengths, suggestions, level of comfort in training others). For each workshop, we calculated average scores for quantitative items and recorded all comments for open-ended questions.

All participants who attended a workshop during the specified period were invited to participate in the follow-up survey, conducted in 2008. We developed a structured telephone interview guide (Appendix) consisting of 43 items that explored use of the NEMS measures, reactions to the NEMS workshop, and training others on the measures. For the purposes of this evaluation, we defined adoption of the NEMS measures as any use, including customization, enumeration (identifying and classifying food outlets), or other planning; data collection, management, or analysis; disseminating or training others on the measures; or use of NEMS measures as a reference for developing another assessment tool. We used archival information (eg, prior communication) when available to supplement survey responses; for 7 NEMS participants who did not respond to the survey, we used archival information to indicate whether they had adopted the NEMS measures. If trainees were not interested in completing a telephone interview, we gave them the option to provide written responses via e-mail.

Telephone interviewers took detailed call notes, which were entered into a database; a second reviewer verified accuracy of data entry. When multiple respondents reported information on the same NEMS project, we grouped their responses for all project-level analyses, including the total number of additional people trained. For quantitative items, we exported data to Microsoft Excel (Microsoft Corporation, Redmond, Washington) and SPSS version 17 (SPSS, Inc, Chicago, Illinois) to calculate descriptive statistics. Chi-squared tests were used to test for differences based on respondents' professional setting and on workshop location, date, and type. For each open-ended question, we developed a matrix to summarize responses, with columns consisting of topics of interest and rows consisting of respondents. Two raters independently completed each matrix and discussed findings to achieve consensus. A primary rater then summarized each matrix to identify major themes, which were reviewed by a second rater.

## Results

### Reach

We have conducted 24 dissemination workshops (6 in 2006, 7 in 2007, 9 in 2008, and 2 in 2009), reaching more than 300 participants from 40 states and the District of Columbia. Additionally, people in 8 foreign countries have attended NEMS workshops or used the measures. Ten workshops were held in Atlanta, Georgia, where the NEMS team was based. At the invitation of local organizers, 14 workshops were held at other locations. In 2008 and 2009, NEMS workshops were included as part of the Built Environment Assessment Training (BEAT) Institute, a week-long program that trains participants to use high-quality measures of nutrition and physical activity environments (www.med.upenn.edu/BEAT).

### Participant characteristics

A total of 173 people attended the 14 workshops included in the follow-up evaluation. Participants' most common professional settings were academic (102 participants) and state or local public health agencies (44 participants). Seventy-four participants attended workshops in Atlanta; 99 attended at other locations. Postcourse evaluations were completed by 154 (94%) of the 164 participants who received these evaluations. A total of 129 respondents (75%) participated in the follow-up survey. There were no significant differences between respondents and nonrespondents in terms of the workshop type, location (Atlanta or off-site), year of training, or professional setting (Table 1).

### Workshop feedback

In postcourse evaluations, participants from all workshops rated the overall workshop an average of 4.81 out of 5, and average ratings for all items were 4.5 or higher. Survey respondents cited fieldwork and practice with the measures, interactive exercises and discussion, structure and organization, and quality of the NEMS team as workshop strengths. Suggestions for improvement included adding more time for discussion; information on data management, cleaning, and analysis; more fieldwork; and more on customizing the measures. Respondents also expressed an interest in learning about how the measures have been used and sharing information among NEMS raters. Participants' comments about the NEMS measures themselves were generally positive, focusing on their ease of use and ability to be customized.

In qualitative responses on the postcourse evaluations, participants who attended the train-the-trainer workshop reported being comfortable with their ability to train others. When follow-up survey respondents who had trained others were asked to rate how prepared they felt on a scale of 1 (not at all prepared) to 5 (extremely prepared), 34 out of 39 respondents replied 4 or 5.

### Use of NEMS measures

A total of 78 respondents reported using the measures. There were no significant differences in use of the measures based on workshop date or location. Respondents who had not used the measures reported barriers such as time, lack of funding, or NEMS not being within the scope of their job. Forty-three respondents reported training a total of 292 additional people on the measures.

Respondents provided information on 46 unique NEMS projects. Seventeen of these projects involved multiple NEMS trainees, who each responded separately to the survey. The mean number of respondents for each project was 1.7 (standard deviation [SD], 1.3; range, 1-8). Respondents used the measures for various purposes including descriptive assessments of diverse nutrition environments (eg, rural, urban, ethnic communities, schools and their surrounding area); comparing availability and pricing of healthy foods in high- and low-income neighborhoods; comparing environmental and individual data; intervention development or evaluation; and exploring the association between nutrition environments and chronic disease rates (Table 2).

Participants used the NEMS measures in 23 states and Washington, DC. Participants most commonly used city limits, county lines, and named neighborhoods to establish the survey area. Twenty-three projects enumerated or rated food outlets; of these, 9 included both stores and restaurants and 14 included stores only. Survey users rated a total of 3,132 food outlets (2,425 stores and 707 restaurants).

Twenty-one projects modified or intend to modify the measures. Users added, revised, or eliminated items to address project-specific needs, such as being regionally or culturally appropriate or addressing specific chronic diseases. For the NEMS-S measures, the most common adaptation was tailoring the measures for Latino/Hispanic populations, for example, adding items such as tortillas. Other projects added items about the store overall (eg, cleanliness, acceptance of vouchers from the Special Supplemental Nutrition Program for Women, Infants, and Children [WIC]). A few projects reported modifying the measurement characteristics of the survey (eg, not collecting data on shelf space). Modifications to the NEMS-R measures were less common and included eliminating the Internet review of menus, only reviewing children's menus, and adding additional items.

Twenty-one projects reported that they had completed data collection; of these, 10 had final results available. Results from NEMS assessments have been reported in 4 peer-reviewed journal articles (21-24), 3 unpublished master's theses (A. Hermstad, 2008; I. Llego Frame, 2007; and L. Wooley, 2006), and several newspaper articles and presentations. NEMS assessments are being used in at least 3 dissertations, not yet published. At the community level, findings have been shared with multiple local audiences, including store and restaurant owners/managers, government and community leaders, community-based organizations, and residents. Respondents reported using NEMS findings to advocate for policy change, promote healthier options at local stores and restaurants, and inform intervention development. Researchers also reported using NEMS as a component of larger studies, for example, to characterize the study setting.

## Discussion

As the focus of obesity research shifts from individual to environmental and policy approaches, valid and reliable measures of the nutrition environment are needed (6,25). Widespread use of such measures may standardize research and provide comparable, high-quality data to inform public policy (6). We developed the NEMS measures to address this need and disseminated them through training workshops, supporting materials, and consultations. The workshops reached a large audience and achieved broad use of the NEMS measures. Almost two-thirds of survey respondents have used the measures in various organizational settings and geographic locations, and for different project purposes. Furthermore, respondents have trained an additional 292 people to use the measures. This number probably underestimates the actual number of additional people who were trained; we know of several NEMS user-led training events for which data are not available, and several training events occurred after we completed data collection. Future study of these secondary training participants may provide useful information about diffusion beyond initial dissemination activities.

Several factors contributed to this successful dissemination. First, the NEMS measures filled an emerging need in both research and community action worlds and continue to do so. Although a number of nutrition environment measures are available (6,7), the NEMS measures are the only resources that have been packaged for distribution and actively communicated through training workshops and Web site resources. The diffusion of innovations model provided a useful framework for dissemination planning. The measures were highly adoptable because they were low in complexity and high in observability and trialability (11). Their relative advantage involved offering an improvement over available options. Also, compatibility was maximized by the developers' approach to dissemination: many participants were surprised by how flexible and supportive we were with respect to modifying the measures. All materials, including the actual measures, were distributed in modifiable formats for participants to tailor. An ongoing, continual process for improving the workshops and measures accelerated their adoption and continued use. The NEMS team was responsive to suggestions for how to support users (eg, providing a data dictionary) and improve future workshops (eg, adding a basic data analysis lesson).

We noted no significant differences in use of the measures based on training date, suggesting that 6 months provided adequate time for participants to begin using the measures. Training location also did not appear to influence use, although off-site trainings were conducted by special request of a host organization. The NEMS-S, or store, measures were more widely used than the NEMS-R restaurant measures (2,425 stores assessed vs 707 restaurants, among evaluation respondents), perhaps because the store measures are easier to use and have fewer areas of ambiguity. The increasing introduction of laws requiring menu labeling in chain restaurants may reduce the complexity of the NEMS-R measures and accelerate their uptake.

Nearly half of respondents who used the measures indicated that they modified or intended to modify them. Whereas such flexibility and modifications are essential to the widespread adoption of the measures in diverse settings, users need to conduct extra developmental research to ensure that adapted versions of NEMS measures retain adequate reliability and validity. Several respondents suggested that the NEMS team facilitate sharing of customized measures, especially those whose reliability and validity have been assessed.

Several limitations should be considered when interpreting evaluation findings. Some selection bias was likely (those who used the measures may have been more likely to respond). The proportion of survey respondents who completed the train-the-trainer component was higher than the proportion for all workshop participants, although not significantly, and we assume that these people would be more likely to use the measures and to train others. We do not assume that the high percentage of use or the number of people trained by survey respondents is representative of all workshop participants. The scope of this evaluation was limited to collection of brief project descriptions; more in-depth exploration of these projects may be an area for future study. When multiple people from the same organization provided project information, it was sometimes difficult to determine whether survey respondents were describing the same project or 2 different projects. In these cases, we assumed that the respondents were describing the same project; using this conservative approach may have caused us to underestimate the number of projects using the measures.

The NEMS training workshops have been an effective dissemination strategy that reached a large number of participants and promoted adoption of the NEMS measures. In January 2010, the NEMS team launched an online instructor-facilitated NEMS training course. This resource should allow greater access; however, future evaluation of the online course compared with the in-person workshop will be important for learning whether users can master the skills without the time and travel requirements of training workshops.

## Figures and Tables

**Table 1:** Characteristics of Nutrition Environment Measures Survey (NEMS) Workshop Participants and Follow-up Survey Respondents

**Characteristics**	Workshop Participants, No. (%) (n = 173)	Survey Respondents, No. (%) (n = 129)
**Type of training attended^a^ **		
Basic	77 (54)	57 (47)
Basic plus train-the-trainer	65 (46)	65 (53)
**Training location **		
Atlanta, Georgia	74 (43)	65 (50)
Off-site	99 (57)	64 (50)
**Training year **		
2006 (6 workshops)	73 (42)	47 (36)
2007 (7 workshops)	94 (54)	76 (59)
2008 (1 workshop)	6 (4)	6 (5)
**Professional setting **		
Academic	102 (59)	76 (59)
State/local public health agency	44 (25)	33 (26)
Community-based organization	8 (5)	5 (4)
Centers for Disease Control and Prevention	13 (8)	11 (9)
Other (eg, local government, private sector)	6 (4)	4 (3)

a Reflects some missing data when type of training was not recorded or reported.

**Table 2:** Examples of Projects Using the Nutrition Environment Measures Surveys (NEMS)

**Project Description**	**Impact**
A community-based organization that aims to reduce overweight/obesity in the local Filipino-American community used a modified NEMS-S survey to assess the availability of healthful options in stores and restaurants serving Filipino-Americans.	The organization partnered with the local Filipino-American newspaper to publish several articles about the assessment. These articles provided information about the local restaurants and encouraged readers to eat healthfully.
A local health department, with funding from the Centers for Disease Control and Prevention, used NEMS as part of a pilot study to improve the nutrition and physical activity environment around a school. After the assessment, they gave stores and restaurants guidelines to help them improve their food choices.	Two locally owned restaurants made adjustments to offer more healthful foods. However, the many chain establishments reported that they have minimal control of their menu and were unable to improve the selections.
As part of the formative research for a childhood obesity prevention program, an academic research team used NEMS to assess stores and restaurants in a rural community. They presented findings to the mayor, city council, and local civic leaders.	To address the mayor's concern that residents would not buy more healthful options, the town sent a survey to all residents; respondents indicated their interest in more healthful options. The research team is now planning to intervene with food distributors and restaurant and store owners.

## Appendix. Telephone Interview Guide

### NEMS Follow-Up Survey

***Interviewer:*** Hello, May I please speak to [participant's name]? This is [your name] calling with the NEMS research team at Emory University. Thank you for taking the time to participate in this interview. As we mentioned in the e-mail you received, we are following up with participants who attended a NEMS training so we can learn more about how the NEMS measures are being used and also ways we can improve our trainings. I will be taking notes based on your response, so if there is a silence, that's why.

1. Which of the following best describes how you have used the NEMS measures since the training? *(Interviewer read the answers)*
  - You have not started to use the NEMS measures, although you may have shared information with colleagues or be thinking about using the measures in the future. → **Go to page 3, Section A**
  - You have begun planning to use the NEMS measures but have not begun data collection. → **Go to page 4, Section B**
  - You have started collecting data with the NEMS measures. → **Go to page 7, Section C**
  - You have completed data collection. → **Go to page 10, Section D**
  *Note to interviewer: Based on response to Question #1, please ask the questions for the section corresponding to participant's answer (* **A-D** *) **and the Training questions section** at the end.*

**A. Not used yet (these participants have done very little since attending their NEMS training)**

2. Are you planning to use the measures in the future?
  □ Yes *→* Could you tell us a little more about your future plans?
  □ No *→* Could you tell us a little more about the reasons you are not going to be doing anything with the NEMS measures?
  [Probe: the measures weren't a good fit, didn't get funding, not in job scope, etc]
  □ Not sure *→* Have you thought about how you might use them?

3. Looking back on the NEMS training you attended, do you have any suggestions for how we could improve the training?
  [Probe — Were there any topics that need to be added or topics covered that needed more explanation?]

***Note to Interviewer: Go to page 14, training questions (Q34)***

**B. Planning stage (these participants are planning to use NEMS but haven't yet collected data)**

4. Can you tell me a little about the project where you are planning to use the NEMS measures?

5. Have you applied for or received any funding or financial support for this project?
  □ Yes
  □ No
  □ In the process
  □ Planning to

6. What do you plan to do with the results of your NEMS assessment?

7. In what geographic area — state(s) and city(ies) or town(s) — will you collect NEMS data?

8. How did you define your survey area? (*if more explanation needed):* What boundaries did you use (for example, city limits, county, named neighborhood)?

9. Have you compiled lists of the food outlets in your survey area and mapped them?
  □ No
  □ Yes *→* How many restaurants do you plan on rating?              How many stores?

10. Do you plan to customize the measures?
  □ No
  □ Maybe
  □ In process ↓
  □ Yes *→* Many people are interested in how others are customizing the measures. Can you please describe how you customized them? Will you share your customized measures with us?
  ***If yes *→**** We will be sending you an e-mail after the interview to remind you to send them to us.

11. Now that you have started planning a NEMS assessment, do you have any suggestions for how we could improve the training?
  [Probe: Are there additional training topics we should add or existing topics that should be covered more in-depth during the training?]

12. a. Would you be interested in attending a NEMS refresher course in the future?
  □ Yes
    □ No
    □ Maybe
  b. Would you be interested in an online refresher course?
  □ Yes
    □ No
    □ Maybe

***Note to Interviewer: Go to page 14, training questions (Q34)***

**C. Data collection has begun**

13. Can you tell me a little about the project where you are using the NEMS measures?

14. Have you applied for or received any funding or financial support for this project?
  □ Yes
  □ No
  □ In process
  □ Maybe

15. What do you plan to do with the results of your NEMS assessment?

16. In what geographic area — state(s) and city(ies) or town(s) — are you collecting NEMS data?

17. How did you define your survey area? (*if more explanation needed):* What boundaries did you use (for example, city limits, county, named neighborhood)?

18. Have you compiled lists of the food outlets in your survey area and mapped them?
  □ No
  □ Yes *→* How many restaurants do you plan on rating?
   How many stores?

19. Did you customize the measures?
  □ No
  □ Yes *→* Many people are interested in how others are customizing the measures. Can you please describe how you customized them? Will you share your customized measures with us?
  ***If yes →*** We will be sending you an e-mail after the interview to remind you to send them to us.

20. Please briefly describe any challenges that you experienced while using the NEMS measures so that we can better prepare raters in the future.

21. Now that you have used the NEMS measures, do you have any suggestions for how we could improve the training?
  [Probe: Are there any additional training topics we should add or existing topics that should be covered more in-depth during the training?]

***Note to Interviewer: Go to page 14, training questions (Q34)***

**D. Data collection has been completed**

22. Can you tell me a little about the project where you used the NEMS measures?

23. Have you applied for or received any funding or financial support for this project?
  □ Yes
  □ No
  □ In process

24. In what geographic area — state(s) and city(ies) or town(s) — did you collect NEMS data?

25. How did you define your survey area? (*if more explanation needed)* What boundaries did you use (for example, city limits, county, named neighborhood)?

26. How many restaurants were rated?
  How many stores were rated?

27. Did you customize the measures?
  □ No
  □ Yes *→* Many people are interested in how others are customizing the measures. Can you please describe how you customized them? Will you share your customized measures with us?
  ***If yes →*** We will be sending you an e-mail after the interview to remind you to send them to us.

28. Please briefly describe any challenges that you experienced while using the NEMS measures, so that we can better prepare raters in the future.

29. Do you have any results to report?
  □ No **(*Skip to Q31*)**
  □ Yes *→* Can you give us a brief summary of your results?
  ***If yes *→**** If you don't mind, we will be sending you an e-mail after the interview to ask you to send us a copy of your results. Would you mind if we share your results with other NEMS trainees? **(Go on to Q30)**

*30. If yes to Q29 ?* What have you already done or what do you intend to do with the results now that you have completed the assessment?

31. *If no to Q29* *→* What do you intend to do with your results once they are available?

32. As part of our effort to learn how the measures work, would you be willing to share your data with us? We will not share your data with anyone outside of our research team without your permission, and we will involve you as collaborators if there are any publications that result from your data.
  □ Yes *→* we will be sending you an e-mail after the interview to remind you to send us your data.
  □ No

33. Now that you have used the NEMS measures, do you have any suggestions for how we could improve the training?
  [Probe: Are there additional training topics we should add or existing topics that should be covered more in-depth during the training?]

***Note to Interviewer: Go to page 14, training questions (Q34)***

**Questions about trainings:**

Now we'll move on to a few questions about whether you have conducted NEMS trainings.

34. Have you trained others to use the NEMS measures? This can include both formal and informal trainings.
  □ Yes *→* Was it a formal NEMS training or on-the-job training?
  □ Formal
    □ On-the-job
  □ No *→* Do you plan to conduct a NEMS training in the future?
  □ Yes **(*Skip to Question 43)***
    □ No **(*Skip to Question 43)***
    □ Maybe **(*Skip to Question 43)***

35. How many people did you train?

36. How would you describe the professions of the people that you trained? *(Interviewer only read list if needed* — *check as many as apply)*
  □ Dietitians or nutrition field
  □ Agricultural extension agents/food security
  □ Public health
  □ Government
  □ Researchers
  □ Students
  □ Community workers

37. Where was the training held (city, state)?

38. How prepared were you for training others on a scale from 1 to 5, with 1 being not at all prepared and 5 being extremely prepared?
  Not at all                                  Extremely prepared
      1            2            3            4            5

39. Did your training include all of the information from the NEMS workshop or did you only train on specific sections?
  □ Full training
  □ Left sections out *→* *If the training was not a full training:* Which sections did you train your raters on? *(Interviewer read the answers; check all that apply)*
  □ Nutrition Overview
    □ Stores
    □ Restaurants
    □ Enumeration
    □ Scoring
    □ Train-the-Trainer
    □ Other (list):

40. Which parts of the NEMS workshop did you feel most prepared for training others on?
  [Probe: For example, menu counting, healthy salads, specific measures, enumeration]

41. Which parts of the NEMS workshop did you not feel entirely prepared for training others on?
  [Probe: for example, menu counting, healthy salads, specific measures, enumeration]

42. Now that you have trained others, is there anything that we could add to the NEMS Workshop that would better prepare participants to train others on the NEMS measures?

43. Do you have any additional comments?
